# *Ex-Vivo* Cytoadherence Phenotypes of *Plasmodium falciparum* Strains from Malian Children with Hemoglobins A, S, and C

**DOI:** 10.1371/journal.pone.0092185

**Published:** 2014-03-19

**Authors:** Jeanette T. Beaudry, Michael A. Krause, Seidina A. S. Diakite, Michael P. Fay, Gyan Joshi, Mahamadou Diakite, Nicholas J. White, Rick M. Fairhurst

**Affiliations:** 1 Laboratory of Malaria and Vector Research, National Institute of Allergy and Infectious Diseases, National Institutes of Health, Bethesda, Maryland, United States of America; 2 Nuffield Department of Clinical Medicine, University of Oxford, Oxford, United Kingdom; 3 Faculty of Medicine, Pharmacy and Odontostomatology, Malaria Research and Training Center, University of Bamako, Bamako, Mali; 4 Biostatistics Research Branch, National Institute of Allergy and Infectious Diseases, National Institutes of Health, Bethesda, Maryland, United States of America; 5 Mahidol-Oxford Tropical Diseases Research Unit, Faculty of Tropical Medicine, Mahidol University, Bangkok, Thailand; University of Copenhagen and Rigshospitalet, Copenhagen, Denmark

## Abstract

Sickle hemoglobin (Hb) S and HbC may protect against malaria by reducing the expression of *Plasmodium falciparum* erythrocyte membrane protein 1 (PfEMP1) on the surface of parasitized red blood cells (RBCs), thereby weakening their cytoadherence to microvascular endothelial cells (MVECs) and impairing their activation of MVECs to produce pathological responses. Therefore, we hypothesized that parasites causing malaria in HbAS or HbAC heterozygotes have overcome this protective mechanism by expressing PfEMP1 variants which mediate relatively strong binding to MVECs. To test this hypothesis, we performed 31 cytoadherence comparisons between parasites from HbAA and HbAS (or HbAC) Malian children with malaria. Ring-stage parasites from HbAA and HbAS (or HbAC) children were cultivated to trophozoites, purified, and then inoculated in parallel into the same wildtype uninfected RBCs. After one cycle of invasion and maturation to the trophozoite stage expressing PfEMP1, parasite strains were compared for binding to MVECs. In this assay, there were no significant differences in the binding of parasites from HbAS and HbAC children to MVECs compared to those from HbAA children (HbAS, fold-change  = 1.46, 95% CI 0.97–2.19, p = 0.07; HbAC, fold-change  = 1.19, 95% CI 0.77–1.84, p = 0.43). These data suggest that *in-vitro* reductions in cytoadherence by HbS and HbC may not be selecting for expression of high-avidity PfEMP1 variants *in vivo*. Future studies that identify PfEMP1 domains or amino-acid motifs which are selectively expressed in parasites from HbAS children may provide further insights into the mechanism of malaria protection by the sickle-cell trait.

## Introduction

For millennia, the significant morbidity and mortality of *Plasmodium falciparum* malaria has selected for red blood cell (RBC) polymorphisms, including sickle hemoglobin (Hb) S, HbC, α-thalassemia, and G6PD deficiency [1]–[10]. These malaria protective polymorphisms have reached high frequencies in tropical areas despite the spectrum of deleterious consequences associated with their homozygous (or hemizygous) states. HbS (β_6_ glu->val) is a balanced polymorphism in which HbAS heterozygotes are protected against both uncomplicated and severe malaria [1]. In contrast, HbC (β_6_ glu->lys) generally affords protection against severe, but not uncomplicated, malaria [1]. Several mechanisms have been proposed to explain this malaria protection, including reduced parasite multiplication rates through reduced invasion, impaired growth, or increased clearance; enhanced innate immunity through inhibition of CD8^+^ T cells and upregulation of heme-oxygenase 1; accelerated acquisition of immunity; and altered host pathogenic mechanisms (reviewed in [11], [12]). One proposed mechanism, which would protect through alterations in host pathogenesis, involves the abnormal display of *P. falciparum* erythrocyte membrane protein 1 (PfEMP1), the parasite's variant surface antigen and cytoadherence ligand, on ‘knobs’ on the surface of parasitized HbAS and HbAC RBCs [13], [14].

Abnormal PfEMP1 display is characterized by (i) reduced PfEMP1 levels, (ii) reduced knob densities, (iii) heterogeneous distributions of PfEMP1 and knobs, and (iv) aberrant – wider and more protuberant – knob morphologies [15]. These perturbations are associated with up to 50% reductions in the cytoadherence of parasitized HbAS and HbAC RBCs [13], [14]. Cytoadherence, the binding of parasitized RBCs to human microvascular endothelial cells (MVECs), enables mature parasites to sequester in the microvessels of most organs and avoid removal from the bloodstream by the spleen [16]. While enabling parasites to multiply to high densities, cytoadherence also contributes to malaria pathogenesis by activating MVECs, leading to release of inflammatory cytokines, upregulation of adhesion receptors, co-sequestration of blood elements (e.g., RBCs, monocytes, and platelets), obstruction of microvessels, and loss of microvascular integrity [17]. Therefore, reductions in PfEMP1-mediated cytoadherence may protect HbAS and HbAC children against malaria by limiting parasite burden and reducing the downstream effects of endothelial cell activation.

The expression of PfEMP1 on parasitized RBCs also leaves parasites vulnerable to detection by the immune system. To avoid this, individual parasites undergo antigenic variation by switching from one clonally-expressed PfEMP1 variant to another [18]. This is accomplished by allelic exclusion of all but one of the parasite's ∼60 *var* genes, which encode PfEMP1 [19]. While repeated and chronic infections with different parasite strains results in the piecemeal acquisition of PfEMP1 variant-specific antibodies [20], the lack of significant strain-transcending immunity and the vast diversity of *var* genes [21], [22] present major obstacles to the development of a PfEMP1-based vaccine. *Var* genes share a two-exon structure, which encodes for a semi-conserved intracellular domain (exon 2) and an extracellular domain (exon 1) comprised of Duffy binding-like domains (DBLs) and cysteine-rich interdomain regions (CIDRs) [19].

Differential affinity of individual DBLs and CIDRs for specific host endothelial receptors (e.g., EPCR in the brain and CD36 in other organs) enables the organ-specific sequestration of parasitized RBCs and influences the clinical presentation and severity of malaria [23], [24]. Recent evidence suggests that particularly virulent subsets of PfEMP1 variants are involved in the pathogenesis of severe malaria syndromes (i.e., respiratory distress, severe anemia, and cerebral malaria) [23], [25]–[27]. Expression of such PfEMP1 variants may provide a selective advantage to parasites by conferring increased cytoadherence or high parasite multiplication rates. Immunity to non-cerebral severe malaria is acquired from 1–2 episodes while immunity to uncomplicated malaria develops over years of repeated and persistent infections [28]. Consistent with these findings, parasites from patients with severe malaria are more frequently recognized by antibodies from immune adults than those from younger patients with uncomplicated malaria [20].

One challenge in defining the mechanisms underlying malaria protection in HbS and HbC children is the difficulty of conducting human *in-vivo* studies to validate *in-vitro* findings. In considering ways to investigate these potential mechanisms, we hypothesized that parasites causing clinical malaria in HbAS and HbAC children must have somehow overcome the malaria protective effects of abnormal PfEMP1 display. One potential mechanism is that parasite populations in HbAS and HbAC children are selected to express particular PfEMP1 variants, which mediate stronger binding to MVECs *in vivo* than those expressed in HbAA children. This process could enable parasites to circumvent the protective effects of abnormal PfEMP1 display and cause malaria symptoms in HbAS and HbAC children. To explore this possibility, we tested whether parasites from HbAS and HbAC children with malaria show increased binding to MVECs *in vitro* than those from HbAA children in Mali.

## Methods

### Study site, participants, and case definitions

*P. falciparum*-infected RBCs and uninfected RBCs were obtained from an ongoing cohort study conducted in three villages (Kenieroba, Fourda, and Bozokin) located 75 km southwest of Bamako, Mali. From these villages, 1514 children aged 0.5–17 years were enrolled over three transmission seasons (2008, 2009, and 2010). All children were typed for Hb polymorphisms (D-10 instrument, BioRad, Hercules, CA), ABO blood group (agglutination assay), α-thalassemia (nested PCR), and presence of the G6PD deficiency allele, G6PD*A- (G202A) (nested PCR), as described [29]. Uncomplicated malaria was defined as axillary temperature >37.5°C and parasite density <100,000/μl counted from thick blood smears. Severe malaria was defined according to WHO criteria [30]. Blood collection and consent process were approved by the Institutional Review Board of the NIAID and the Ethics Committee of the Faculty of Medicine, Pharmacy, and Odontostomatology at the University of Bamako. Informed consent was obtained from adults or the parent/guardian of children. Written informed consent was obtained from those adults who could read the informed consent form in French. Oral informed consent was obtained in the local language from those adults who could not read French. In this case, oral consent was documented by the adult's thumbprint and the study physician's signature, and the consent process witnessed by a third-party, French-literate individual from the community. This study is registered at Clinicaltrials.gov (NCT00669084).

### Parasite culture

Venous blood samples from children with malaria were collected in sodium heparin Vacutainers (Becton-Dickinson, Franklin Lakes, NJ), and the RBCs washed three times in RPMI 1640 (Life Technologies, Grand Island, NY). Uninfected ‘wildtype’ donor RBCs [i.e., HbAA, normal αα/αα genotype, and no G6PD*(A-) allele] from healthy volunteers were similarly collected, washed, resuspended at 50% hematocrit, and stored at 4°C for up to 3 days before use. Ring-stage parasites from HbAA, HbAS, and HbAC children with malaria (**Table 1**) were cultured at 1 or 2% hematocrit in complete medium [RPMI 1640 supplemented with 2 mg/ml sodium bicarbonate, 50 μg/ml gentamicin, and 0.5% Albumax II (Life Technologies)]. Parasites were cultured for ∼24 h to trophozoites in 0.2-μm-vented flasks (Corning Inc., Corning, NY) in a humidified 5% CO_2_ environment at 37°C. Trophozoite-infected RBCs were then magnetically purified (Miltenyi Biotec, Auburn, CA) and inoculated into wildtype RBCs at 2.5% hematocrit. After invasion of uninfected RBCs was confirmed by the appearance of new ring-stage parasites, the hematocrit was decreased to 0.8%. Once parasites matured to trophozoites, they were assayed for cytoadherence to MVECs in parallel with one or more other parasite isolates that were simultaneously cultivated, purified, and inoculated into the same wildtype RBCs.

**Table 1:** Characteristics of Malian HbAA, HbAS, and HbAC children from which parasite isolates were obtained.

*All* samples used in comparisons										
		α-thalassemia			G6PD*A- (G202A)					
	n	WT	HET	ND	ABS	HET	HEM	Age	Pf den	% sev
**HbAA**	31	87.1	9.7	3.2	87.1	3.2	9.7	6.2	25200	0
**HbAS**	16	68.8	25.0	6.3	87.5	12.5	0	4.9	22125	12.5
**HbAC**	15	60.0	40.0	0	80.0	20.0	0	5.9	32025	13.3

*Unique* samples used in comparisons										
		α-thalassemia			G6PD*A- (G202A)					
	n	WT	HET	ND	ABS	HET	HEM	Age	Pf den	% sev
**HbAA**	25	84.0	12.0	4.0	84.0	4.0	12.0	6.0	19200	0
**HbAS**	15	66.7	26.7	6.7	86.7	13.3	0	5.2	22050	13.3
**HbAC**	12	58.3	41.7	0	83.3	16.7	0	5.9	26288	8.3

The number of samples (n), proportion of samples with α-thalassemia (wildtype, WT; heterozygous, HET; not determined, ND) and G6PD*A- (G202A) (absent, ABS; heterozygous, HET; hemizygous, HEM) genotypes, mean age (years), median parasite density (/μl), and proportion of severe malaria cases are shown for *all* HbAA, HbAS, and HbAC samples used in comparisons, and *unique* HbAA, HbAS, and HbAC samples used in comparisons. *All* samples (n = 62) include 10 parasite strains that were used in multiple comparisons, while *unique* samples include 52 parasite strains that were used in single comparisons. Three *unique* samples (2 HbAS and 1 HbAC) were classified as severe since they met one or more of these criteria: cessation of eating/drinking, repetitive vomiting, or prostration.

### Cytoadherence assay

Human dermal MVECs (HMVECs-d, Lonza, Walkersville, MD) were grown in the manufacturer's EGM-2MV media for up to 5 passages and seeded into 8-well LabTak CC2-coated chamber slides (Nalge Nunc International, Rochester, NY) to achieve ∼30% confluency. Trophozoite-infected RBCs were magnetically purified and adjusted to 3–5% parasitemia (with equal parasitemias for samples compared on the same slide) and 0.5% hematocrit using uninfected RBCs in binding medium (BM; RPMI 1640, 0.5% BSA). Parasitized RBC suspensions were added to MVEC-coated chamber slides (200 μl/well) and incubated on an orbital shaker (100 rpm) for 1 h at room temperature. Whenever possible, each parasite isolate was tested for binding in duplicate wells on the same slide. After the parasitized RBC suspensions were removed from each well, the gasket was detached from the slide. The slide was then washed by dipping horizontally in BM four times, fixed in 2% glutaraldehyde overnight at room temperature, and stained in 10% Giemsa for 30 min. The number of parasitized RBCs bound to ∼350 MVECs in each well was counted, and data expressed as number of parasitized RBCs per MVEC. Duplicate comparisons were made whenever sufficient numbers of parasites were available. Seventy-six percent (25/33) of comparisons were performed in duplicate, 50% of which had a ratio of replicate 1∶2 between 0.80 and 1.3 (range 0.5–2.5).

### Statistical analysis

An overdispersed Poisson regression was used to model the effect of Hb type (HbAS and HbAC) and age (≤5 and >5 years) on the binding of parasitized RBCs to MVECs, controlling for any ‘slide’ and ‘location within slide’ effects in our data set and for any multiple comparisons done with an individual parasite isolate. Analyses were done in Proc GLMMIX in SAS Version 9.2.

## Results

**Figure 1:**
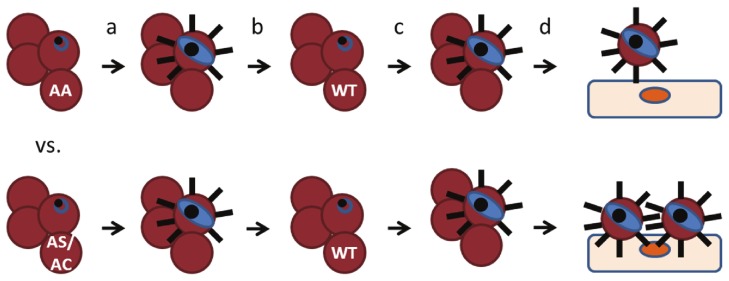
Schematic representation of the cytoadherence assay. Ring-stage parasites from HbAA and HbAS (or HbAC) children were cultured to trophozoites (a), and then purified by magnetic column and inoculated into wildtype donor RBCs (b). After invasion and maturation to trophozoites expressing PfEMP1 (c), parasites from HbAS or HbAC children were compared for binding to MVECs in parallel with those from HbAA children (d).

**Figure 2:**
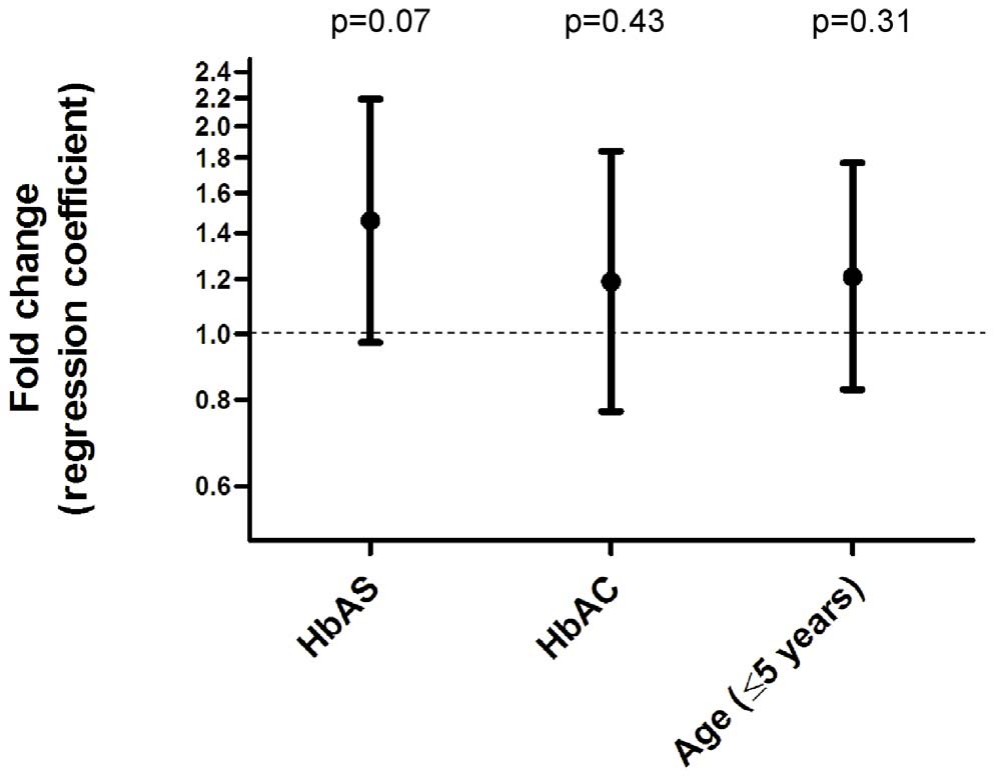
Relationship between cytoadherence, Hb type, and host age. A Poisson regression model was constructed to examine the effect of Hb type and host age on the cytoadherence of parasitized RBCs to MVECs. Fold-changes and 95% CIs of the relative binding compared to parasites from HbAA (for HbAS and HbAC) children and parasites from >5-year-old (for ≤5-year-old) children are indicated.

In children with malaria, we hypothesized that HbAS and HbAC select for parasites with stronger binding to MVECs than HbAA. To test this hypothesis, ring-stage parasites obtained directly from HbAA, HbAS and HbAC children (**Table 1**) were cultured *ex vivo* to the trophozoite stage, magnetically purified, and inoculated into the same wildtype donor RBCs (**Figure 1**). Once parasites invaded and matured to trophozoites expressing PfEMP1, we compared the cytoadherence of parasites from HbAS or HbAC children to parasites from HbAA children, tested in parallel. Parasites from HbAS children showed increased binding to MVECs compared to those from HbAA children, but this increase was not significant (fold-change  = 1.46, 95% CI 0.97–2.19, n = 16, p = 0.07) (**Figure 2** **,** **Table 2**). Parasites from HbAC children clearly showed no difference in binding (fold-change  = 1.19, 95% CI 0.77–1.84, n = 15, p = 0.43) (**Figure 2** **,** **Table 2**). Since age, a surrogate of immunity in areas of high malaria transmission, influences the expressed PfEMP1 repertoire, we included it as a categorical covariate. Parasites from younger children (≤5 years) showed no difference in binding compared to those from older children (>5 years) (fold-change  = 1.21, 95% CI 0.83–1.77, n = 31, p = 0.31) (**Figure 2**).

**Table 2:** Relative cytoadherence of parasitized HbAA, HbAS, and HbAC RBCs to MVECs.

HbAS:HbAA comparisons						
Slide #	Year	ID	pRBC	MVEC	pRBC/MVEC	Ratio
1	2008	AS1	1462	1216	1.20	1.41
		AA1	1054	1236	0.85	
2	2008	AS2	758	627	1.21	1.76
		AA2	427	620	0.69	
3	2009	**AS3**	12279	709	17.32	1.68
		AA3	7353	712	10.33	
4	2009	**AS3**	7714	704	10.96	6.73
		AA4	1147	704	1.63	
5	2009	AS4	1687	707	2.39	0.63
		AA5	2672	705	3.79	
6	2009	AS5	1180	729	1.62	1.26
		**AA6**	930	725	1.28	
6	2009	AS6	3773	703	5.37	4.18
		**AA6**	930	725	1.28	
6	2009	AS7	2589	703	3.68	2.87
		**AA7**	930	725	1.28	
7	2009	AS8	4392	708	6.20	1.18
		AA7	3864	733	5.27	
8	2009	AS9	696	727	0.96	0.33
		AA8	2125	740	2.87	
9	2009	AS10	3003	711	4.22	3.78
		AA9	795	711	1.12	
10	2010	AS11	4870	730	6.67	1.46
		AA10	3661	799	4.58	
11	2010	AS12	3818	871	4.38	0.98
		AA11	3712	830	4.47	
12	2010	AS13	1759	712	2.247	0.58
		AA12	2880	747	3.86	
13	2010	AS13	3180	727	4.37	0.66
		AA12	4768	728	6.55	
14	2010	AS14	6033	727	8.30	2.43
		AA13	2412	706	3.42	
15	2010	AS15	2929	714	4.10	1.64
		AA14	1917	767	2.50	

HbAC:HbAA comparisons						
16	2008	AC1	1581	600	2.64	2.20
		AA15	360	300	1.20	
17	2008	AC2	712	608	1.17	1.75
		AA16	402	600	0.67	
18	2008	AC3	307	300	1.02	1.72
		AA17	179	300	0.60	
19	2008	AC4	844	300	2.81	2.36
		AA18	715	600	1.19	
20	2008	AC5	244	302	0.81	1.58
		**AA19**	153	300	0.51	
20	2008	AC6	922	625	1.48	2.89
		**AA19**	153	300	0.51	
20	2008	AC7	738	604	1.22	2.40
		**AA19**	153	300	0.51	
21	2008	AC8	1089	600	1.82	1.04
		AA20	1048	600	1.75	
22	2009	**AC9**	4452	735	6.06	0.82
		AA3	5255	713	7.37	
4	2009	AC9	2042	354	5.77	3.54
		AA4	1147	704	1.63	
23	2009	**AC10**	9197	706	13.03	5.00
		AA21	1825	701	2.60	
23	2009	AC10	9197	706	13.03	3.37
		AA22	2329	704	3.31	
24	2009	**AC10**	12512	707	17.70	3.05
		AA22	4255	732	5.81	
25	2009	AC11	1603	720	2.23	0.46
		AA23	3560	715	4.84	
26	2009	**AC12**	2139	700	3.06	0.60
		AA24	1789	350	5.11	
27	2010	**AC12**	1970	747	2.64	0.35
		AA25	5328	707	7.54	

Over three transmission seasons (2008, 2009, and 2010), a total of 31 cytoadherence comparisons were performed between parasites from HbAA and HbAS (or HbAC) children by inoculating them into wild-type donor RBCs and assaying their binding to MVECs in parallel. The ratio of parasitized RBCs (pRBC) bound per MVEC (pRBC/MVEC) was calculated for each sample tested. The pRBC/MVEC ratio from the HbAS- or HbAC-derived parasite in each comparison was then normalized to that of the HbAA-derived parasite from the same comparison to give a measure of the relative cytoadherence. Two comparisons (AS13 *vs.* AA12 and AC10 *vs.* AA22) were performed over multiple slides, as shown. Also, several samples (indicated in bold) were used in multiple comparisons.

## Discussion

We hypothesized that *P. falciparum* parasites in HbAS and HbAC children with malaria overcome abnormal PfEMP1 display by expressing PfEMP1 variants which bind relatively strongly to MVECs. In our study, HbAS, HbAC, and young age were not significantly associated with increased binding to MVECs. However, these data require confirmation as they were obtained from a limited number of comparisons between parasites from HbAS, HbAC, and HbAA children. Working in a cohort of ∼1350 children (15.3% HbS, 7.3% HbC) in an area of intense seasonal transmission for 3 years, we were able to make only 16 comparisons between HbAS and HbAA samples, and only 15 comparisons between HbAC and HbAA samples. The number of comparisons was limited by several factors. First, high numbers of parasites were required to conduct the *ex-vivo* cytoadherence assay before parasites switched PfEMP1 variants (i.e., the parasite isolates could not be adapted and expanded in culture). Second, the assay required that parasites from HbAS (or HbAC) children mature synchronously in culture with those from HbAA children, which did not always occur. Third, HbAS conferred significant protection against malaria in our cohort (unpublished data) and so fewer parasite isolates were available from HbAS children. Additional factors limited data analysis. For example, more comparisons would have enabled us to include age as a continuous variable, providing finer resolution of age effects on cytoadherence. Also, 42% of comparisons between HbAS (or HbAC) and HbAA samples may have been confounded by co-inherited RBC polymorphisms (e.g., α-thalassemia and G6PD deficiency) (**Table 1**), which could also affect cytoadherence [31]. Finally, comparisons of relative cytoadherence may have been influenced by different batches of MVECs, which can vary in their ability to bind parasitized RBCs.

It remains unclear whether our results reflect the limitations described above or to an actual lack of difference in binding phenotypes for parasites from HbAA and HbAS children – especially since parasites from HbAS children trended towards increased binding to MVECs, but failed to reach significance. However, the lack of statistical significance in both sets of comparisons suggests that any cytoadherence-dependent mechanism is likely working in concert with other factors (e.g., PfEMP1-specific IgG) to prevent the development of uncomplicated and severe malaria syndromes. Additional studies that are powered to address some of these limitations are therefore warranted since the results from such studies would have two significant implications. First, they would provide insight into whether abnormal PfEMP1 display is working *in vivo* to modulate the expressed *var* gene repertoire of parasite populations. Second, if a subset of high-avidity PfEMP1 variants are, indeed, being selected in HbAS children, they might offer an opportunity to identify “virulent” PfEMP1 variants and thus aid the discovery of putative “pathogenicity motifs” (e.g., particular PfEMP1 domains, or conserved amino-acid residues within domains) that confer high-avidity binding phenotypes. While such motifs may only cause uncomplicated malaria in older children with HbAS, for example, they may be responsible for causing severe malaria in younger HbAA children who lack both immune- and hemoglobinopathy-mediated resistance to this disease. Such an approach would complement efforts to characterize PfEMP1 variants expressed in children with severe malaria. Since parasites from these children show increased binding to CD36 and ICAM-1, the PfEMP1 variants causing uncomplicated malaria in HbAS children might share features with those causing severe malaria in other children.

The results of our study suggest that our particular binding assay detected no clear differences between the cytoadherence of parasites from HbAA, HbAS, and HbAC children to MVECs. Although further studies that are adequately powered to account for age, severity of malaria episode, and co-inherited RBC polymorphisms are needed, our initial findings provide useful insights into the relationship between Hb types and PfEMP1 variants *in vivo*. Testing the binding of parasites from HbAS, HbAC, and HbAA children with malaria to individual receptors (e.g., CD36, ICAM-1, gC1qR, EPCR) as well as to MVECs derived from brain or lung may help to further clarify the binding phenotypes of these parasites. Furthermore, identification of “virulent” PfEMP1 domains associated with increased binding to MVECs may inform the rational design of PfEMP1-based vaccines against severe malaria.
